# Supplementary material for: Effects of a Prenatal Lifestyle Intervention in Routine Care on Maternal Health Behaviour in the First Year Postpartum—Secondary Findings of the Cluster-Randomised GeliS Trial
Source: Nutrients. 2021 Apr 15;13(4):1310. doi: 10.3390/nu13041310 (PMC8071441; doi:10.3390/nu13041310)
Supplement: Supplementary file 1 [file nutrients-13-01310-s001.zip › nutrients-1169302-supplementary.pdf]

Table S1 Healthy Eating Index in subgroups of the intervention and control groups.

	Intervention Group		Control Group		n [a]	Adjusted Effect Size [b] (95% CI)	Adjusted *p* Value [b]
	n	Mean ± SD	n	Mean ± SD			
**T1pp**							
**Pre-pregnancy age category**							
Age 18–25 years	107	53.83 ± 9.19	119	51.25 ± 7.72	214	0.08 (-1.25, 1.41)	0.908
Age 26–35 years	687	55.84 ± 8.17	650	55.12 ± 8.35	1249	0.65 (-0.44, 1.73)	0.243
Age 36–43 years	104	57.75 ± 8.46	123	56.56 ± 8.27	213	0.62 (0.02, 1.22)	0.044
**Pre-pregnancy BMI category**							
BMI 18.5–24.9 kg/m^2	584	56.08 ± 8.29	595	55.25 ± 8.41	1100	0.48 (-0.69, 1.64)	0.421
BMI 25.0–29.9 kg/m^2	217	55.49 ± 8.25	189	54.10 ± 8.37	379	1.28 (0.92, 1.64)	<0.001
BMI 30.0–40.0 kg/m^2	97	54.98 ± 9.12	109	53.63 ± 8.10	197	0.95 (-0.73, 2.63)	0.266
**Educational level [c]**							
General secondary school	124	52.93 ± 8.36	143	50.93 ± 8.42	251	1.37 (-0.59, 3.33)	0.171
Intermediate secondary school	388	54.97 ± 8.12	371	54.38 ± 7.99	707	0.22 (-0.59, 1.03)	0.593
High school	386	57.60 ± 8.25	378	56.69 ± 8.21	718	0.74 (-0.30, 1.77)	0.162
**Parity**							
Primiparous	574	55.47 ± 8.30	487	54.05 ± 8.36	993	0.89 (-0.30, 2.09)	0.142
Multiparous	324	56.44 ± 8.48	406	55.72 ± 8.32	683	0.15 (-0.10, 0.40)	0.248
**T2pp**							
**Pre-pregnancy age category**							
Age 18–25 years	88	54.77 ± 8.80	94	52.31 ± 9.47	171	1.07 (-0.47, 2.62)	0.172
Age 26–35 years	605	56.30 ± 8.78	572	55.29 ± 8.27	1117	0.84 (0.18, 1.51)	0.013
Age 36–43 years	98	57.03 ± 8.76	110	56.59 ± 7.08	197	1.01 (-0.61, 2.64)	0.222
**Pre-pregnancy BMI category**							
BMI 18.5–24.9 kg/m^2	513	56.55 ± 8.90	506	55.44 ± 8.55	966	0.78 (-0.13, 1.69)	0.093
BMI 25.0–29.9 kg/m^2	186	55.59 ± 7.85	178	54.27 ± 8.02	343	1.29 (-0.03, 2.61)	0.055
BMI 30.0–40.0 kg/m^2	92	55.65 ± 9.85	93	54.88 ± 7.62	176	0.25 (-2.51, 3.00)	0.861
**Educational level [c]**							
General secondary school	96	53.00 ± 9.08	117	51.73 ± 8.82	203	0.50 (-0.26, 1.27)	0.200
Intermediate secondary school	347	55.57 ± 7.93	322	54.21 ± 8.27	633	1.35 (0.78, 1.91)	<0.001
High school	347	57.77 ± 9.22	337	57.15 ± 7.69	648	0.84 (-0.58, 2.26)	0.245
**Parity**							
Primiparous	499	55.98 ± 8.92	419	54.83 ± 8.37	869	0.71 (-0.31, 1.73)	0.175
Multiparous	292	56.64 ± 8.55	358	55.43 ± 8.29	616	1.07 (0.33, 1.82)	0.005

Abbreviations: BMI: body mass index; T1pp: assessment 6-8 weeks postpartum; T2pp: assessment one year postpartum; SD: standard deviation; CI: confidence interval. [a] The total of participant numbers varies due to the applied covariates. [b] Linear regression models fit using generalised estimating equations adjusted for pre-pregnancy BMI category, age, parity, baseline assessment and time interval between questionnaire completion date and birth date of the child. [c] General secondary school: general school which is completed through year 9; intermediate secondary school: vocational secondary school which is completed through year 10; high school: academic high school which is completed through year 12 or 13.

Table S2 Total physical activity in subgroups of the intervention and control groups.

	Intervention Group		Control Group		n [a]	Adjusted Effect Size [b] (95% CI)	Adjusted *p* Value [b]
	n	Mean ± SD	n	Mean ± SD			
**T1pp**							
**Pre-pregnancy age category**							
Age 18–25 years	97	190.99 ± 64.86	116	202.95 ± 73.21	193	-16.28 (-32.09, -0.47)	0.044
Age 26–35 years	654	177.97 ± 64.41	627	180.57 ± 68.95	1146	1.18 (-3.33, 5.68)	0.608
Age 36–43 years	102	174.28 ± 59.20	119	176.33 ± 60.65	199	-5.01 (-14.71, 4.68)	0.311
**Pre-pregnancy BMI category**							
BMI 18.5–24.9 kg/m²	555	179.11 ± 65.10	575	182.43 ± 68.46	1013	-4.18 (-8.30, -0.06)	0.047
BMI 25.0–29.9 kg/m²	206	176.30 ± 60.44	184	181.44 ± 69.28	349	-2.03 (-8.43, 4.37)	0.534
BMI 30.0–40.0 kg/m²	92	184.43 ± 64.86	104	189.00 ± 70.27	176	0.18 (-11.41, 11.77)	0.975
**Educational level [c]**							
General secondary school	116	185.30 ± 78.95	137	198.96 ± 79.58	214	-17.54 (-33.64, -1.44)	0.033
Intermediate secondary school	369	177.39 ± 61.04	357	182.31 ± 70.08	654	0.06 (-4.24, 4.37)	0.977
High school	368	178.65 ± 61.55	368	177.71 ± 62.35	670	1.43 (-6.83, 9.68)	0.735
**Parity**							
Primiparous	541	176.30 ± 61.01	467	182.27 ± 68.07	911	-8.59 (-13.59, -3.59)	0.001
Multiparous	312	183.71 ± 68.60	396	183.88 ± 69.76	627	4.92 (-0.24, 10.07)	0.062
**T2pp**							
**Pre-pregnancy age category**							
Age 18–25 years	78	205.59 ± 70.85	87	207.26 ± 78.15	144	3.22 (-19.70, 26.13)	0.783
Age 26–35 years	588	188.45 ± 65.11	545	186.44 ± 69.92	1016	2.86 (-6.07, 11.79)	0.530
Age 36–43 years	88	181.49 ± 53.59	105	180.78 ± 67.41	178	2.35 (-12.51, 17.22)	0.756
**Pre-pregnancy BMI category**							
BMI 18.5–24.9 kg/m²	471	190.64 ± 62.10	480	186.47 ± 70.81	867	5.14 (-2.32, 12.60)	0.177
BMI 25.0–29.9 kg/m²	166	185.61 ± 64.83	168	193.16 ± 67.97	308	-6.58 (-17.75, 4.58)	0.248
BMI 30.0–40.0 kg/m²	87	190.35 ± 77.36	89	187.25 ± 76.67	163	11.05 (-1.99, 24.08)	0.097
**Educational level [c]**							
General secondary school	86	205.93 ± 80.21	109	201.76 ± 79.51	166	-1.25 (-24.56, 22.06)	0.916
Intermediate secondary school	320	187.99 ± 63.17	305	192.53 ± 77.18	581	0.06 (-13.36, 13.48)	0.993
High school	317	186.42 ± 61.02	323	179.28 ± 59.79	591	4.29 (-5.04, 13.62)	0.368
**Parity**							
Primiparous	460	189.05 ± 65.13	397	187.43 ± 66.78	782	1.22 (-10.58, 13.01)	0.840
Multiparous	264	190.16 ± 63.99	340	188.86 ± 78.45	556	6.55 (2.10, 11.00)	0.004

Abbreviations: BMI: body mass index; T1pp: assessment 6-8 weeks postpartum; T2pp: assessment one year postpartum; SD: standard deviation; CI: confidence interval. [a] The total of participant numbers varies due to the applied covariates. [b] Linear regression models fit using generalised estimating equations adjusted for pre-pregnancy BMI category, age, parity, baseline assessment and time interval between questionnaire completion date and birth date of the child. [c] General secondary school: general school which is completed through year 9; intermediate secondary school: vocational secondary school which is completed through year 10; high school: academic high school which is completed through year 12 or 13.

Table S3 Meeting physical activity recommendations in subgroups of the intervention and control groups.

	Intervention Group		Control Group		n [a]	Adjusted OR [b] (95% CI)	Adjusted *p* Value [b]
	n	%	n	%			
**T1pp**							
**Pre-pregnancy age category**							
Age 18–25 years	57/103	55.3%	58/118	49.2%	214	1.28 (1.01, 1.62)	0.044
Age 26–35 years	358/676	53.0%	308/645	47.8%	1259	1.13 (0.99, 1.29)	0.067
Age 36–43 years	51/105	48.6%	49/124	39.5%	218	1.12 (0.87, 1.44)	0.372
**Pre-pregnancy BMI category**							
BMI 18.5–24.9 kg/m²	315/576	54.7%	283/592	47.8%	1111	1.20 (1.05, 1.37)	0.007
BMI 25.0–29.9 kg/m²	104/213	48.8%	86/190	45.3%	387	1.08 (0.75, 1.55)	0.686
BMI 30.0–40.0 kg/m²	47/95	49.5%	46/106	43.4%	193	1.16 (0.95, 1.43)	0.149
**Educational level [c]**							
General secondary school	54/121	44.6%	62/143	43.4%	254	1.09 (0.90, 1.31)	0.388
Intermediate secondary school	206/384	53.6%	169/366	46.2%	715	1.16 (0.96, 1.40)	0.130
High school	206/379	54.4%	184/378	48.7%	722	1.21 (0.91, 1.59)	0.185
**Parity**							
Primiparous	337/566	59.5%	260/482	53.9%	1004	1.26 (1.05, 1.51)	0.014
Multiparous	129/318	40.6%	155/406	38.2%	687	0.96 (0.85, 1.09)	0.529
**T2pp**							
**Pre-pregnancy age category**							
Age 18–25 years	48/82	58.5%	49/89	55.1%	165	0.84 (0.48, 1.47)	0.536
Age 26–35 years	344/579	59.4%	311/556	55.9%	1108	1.12 (1.05, 1.20)	0.001
Age 36–43 years	47/95	49.5%	56/107	52.3%	194	0.59 (0.27, 1.28)	0.180
**Pre-pregnancy BMI category**							
BMI 18.5–24.9 kg/m²	295/488	60.5%	286/490	58.4%	949	0.95 (0.70, 1.28)	0.720
BMI 25.0–29.9 kg/m²	100/179	55.9%	83/172	48.3%	342	1.47 (1.10, 1.96)	0.009
BMI 30.0–40.0 kg/m²	44/89	49.4%	47/91	51.6%	176	0.71 (0.47, 1.09)	0.122
**Educational level [c]**							
General secondary school	46/89	51.7%	48/111	43.2%	196	1.09 (0.81, 1.47)	0.572
Intermediate secondary school	189/333	56.8%	162/311	52.1%	628	1.04 (0.68, 1.60)	0.857
High school	203/333	61.0%	206/330	62.4%	642	0.85 (0.60, 1.21)	0.372
**Parity**							
Primiparous	296/477	62.1%	265/405	65.4%	858	0.83 (0.67, 1.03)	0.094
Multiparous	143/279	51.3%	151/348	43.4%	609	1.44 (1.23, 1.68)	<0.001

Abbreviations: BMI: body mass index; T1pp: assessment 6-8 weeks postpartum; T2pp: assessment one year postpartum; OR: odds ratio. [a] The total of participant numbers varies due to the applied covariates. [b] Binary logistic regression models fit using generalised estimating equations adjusted for pre-pregnancy BMI category, age, parity, baseline assessment and time interval between questionnaire completion date and birth date of the child. [c] General secondary school: general school which is completed through year 9; intermediate secondary school: vocational secondary school which is completed through year 10; high school: academic high school which is completed through year 12 or 13.

Table S4 Sedentary activity in subgroups of the intervention and control groups.

	**Intervention Group**		**Control Group**		**n [a]**	**Adjusted Effect Size [b] (95% CI)**	**Adjusted *p* Value [b]**
	**n**	**Mean ± SD**	**n**	**Mean ± SD**			
**T1pp**							
**Pre-pregnancy age category**							
Age 18–25 years	107	11.22 ± 11.63	120	13.29 ± 11.70	208	-3.09 (-5.44, -0.74)	0.010
Age 26–35 years	690	10.17 ± 9.96	653	10.58 ± 10.22	1243	-0.62 (-1.54, 0.30)	0.185
Age 36–43 years	106	8.31 ± 8.12	125	9.08 ± 9.16	213	-0.92 (-2.32, 0.48)	0.198
**Pre-pregnancy BMI category**							
BMI 18.5–24.9 kg/m²	589	9.63 ± 9.30	598	10.60 ± 10.37	1092	-1.41 (-2.66, -0.16)	0.027
BMI 25.0–29.9 kg/m²	216	10.90 ± 11.42	192	11.02 ± 9.93	380	0.16 (-1.49, 1.81)	0.853
BMI 30.0–40.0 kg/m²	98	10.95 ± 10.55	109	10.88 ± 10.96	192	0.14 (-1.22, 1.49)	0.844
**Educational level [c]**							
General secondary school	122	9.84 ± 10.44	143	13.36 ± 12.68	236	-3.36 (-5.73, -1.00)	0.005
Intermediate secondary school	392	9.83 ± 9.81	373	11.03 ± 10.52	703	-2.15 (-3.10, -1.21)	<0.001
High school	389	10.41 ± 10.04	382	9.46 ± 8.91	725	0.93 (0.29, 1.57)	0.004
**Parity**							
Primiparous	573	11.30 ± 10.82	489	12.41 ± 11.26	989	-1.13 (-2.37, 0.11)	0.075
Multiparous	330	7.96 ± 7.96	410	8.71 ± 8.71	675	-0.69 (-1.98, 0.60)	0.295
**T2pp**							
**Pre-pregnancy age category**							
Age 18–25 years	81	8.90 ± 7.96	88	9.87 ± 8.87	152	-0.27 (-2.25, 1.70)	0.787
Age 26–35 years	595	6.99 ± 5.65	561	7.36 ± 6.03	1099	-0.45 (-1.05, 0.16)	0.146
Age 36–43 years	97	6.37 ± 5.25	110	7.25 ± 7.00	195	-0.99 (-2.22, 0.25)	0.118
**Pre-pregnancy BMI category**							
BMI 18.5–24.9 kg/m²	500	6.67 ± 5.30	495	7.33 ± 6.45	938	-0.67 (-1.38, 0.03)	0.061
BMI 25.0–29.9 kg/m²	183	7.62 ± 6.05	173	7.91 ± 5.89	338	-0.11 (-0.48, 0.25)	0.547
BMI 30.0–40.0 kg/m²	90	8.48 ± 8.25	91	8.79 ± 8.43	170	-1.06 (-1.82, -0.30)	0.006
**Educational level [c]**							
General secondary school	91	8.23 ± 7.06	112	9.21 ± 8.53	184	-1.48 (-3.02, 0.06)	0.060
Intermediate secondary school	342	7.74 ± 6.30	313	7.82 ± 6.30	620	-0.51 (-1.22, 0.20)	0.158
High school	339	6.44 ± 5.07	334	6.94 ± 6.04	641	-0.26 (-0.93, 0.41)	0.443
**Parity**							
Primiparous	489	7.52 ± 6.46	406	8.08 ± 6.84	844	-0.52 (-1.05, 0.01)	0.053
Multiparous	284	6.41 ± 4.78	353	7.13 ± 6.29	602	-0.86 (-1.41, -0.32)	0.002

Abbreviations: BMI: body mass index; T1pp: assessment 6-8 weeks postpartum; T2pp: assessment one year postpartum; SD: standard deviation; CI: confidence interval. [a] The total of participant numbers varies due to the applied covariates. [b] Linear regression models fit using generalised estimating equations adjusted for pre-pregnancy BMI category, age, parity, baseline assessment and time interval between questionnaire completion date and birth date of the child. [c] General secondary school: general school which is completed through year 9; intermediate secondary school: vocational secondary school which is completed through year 10; high school: academic high school which is completed through year 12 or 13.

Table S5 Smoking in subgroups of the intervention and control groups.

	Intervention Group		Control Group		n [a]	Adjusted OR [b] (95% CI)	Adjusted *p* Value [b]
	n	%	n	%			
**T1pp**							
**Pre-pregnancy age category**							
Age 18–25 years	17/109	15.6%	28/121	23.1%	223	0.38 (0.21, 0.68)	0.001
Age 26–35 years	39/692	5.6%	49/661	7.4%	1289	0.79 (0.67, 0.94)	0.009
Age 36–43 years	8/104	7.7%	11/127	8.7%	221	0.29 (0.09, 0.95)	0.041
**Pre-pregnancy BMI category**							
BMI 18.5–24.9 kg/m^2	43/590	7.3%	49/604	8.1%	1137	0.76 (0.60, 0.96)	0.019
BMI 25.0–29.9 kg/m^2	11/218	5.0%	22/194	11.3%	395	0.34 (0.16, 0.70)	0.004
BMI 30.0–40.0 kg/m^2	10/97	10.3%	17/112	15.2%	201	0.23 (0.02, 2.39)	0.218
**Educational level [c]**							
General secondary school	22/124	17.7%	37/146	25.3%	258	0.63 (0.38, 1.03)	0.063
Intermediate secondary school	34/392	8.7%	42/377	11.1%	734	0.51 (0.40, 0.66)	<0.001
High school	8/389	2.1%	9/386	2.3%	741	1.17 (0.74, 1.85)	0.491
**Parity**							
Primiparous	37/576	6.4%	40/493	8.1%	1026	0.50 (0.40, 0.62)	<0.001
Multiparous	27/329	8.2%	48/417	11.5%	707	0.55 (0.28, 1.06)	0.074
**T2pp**							
**Pre-pregnancy age category**							
Age 18–25 years	19/88	21.6%	28/94	29.8%	176	0.62 (0.32, 1.20)	0.156
Age 26–35 years	73/600	12.2%	71/568	12.5%	1137	1.00 (0.89, 1.11)	0.955
Age 36–43 years	11/97	11.3%	10/109	9.2%	199	5.08 (0.79, 32.82)	0.088
**Pre-pregnancy BMI category**							
BMI 18.5–24.9 kg/m^2	63/509	12.4%	65/504	12.9%	984	0.82 (0.70, 0.96)	0.017
BMI 25.0–29.9 kg/m^2	28/184	15.2%	26/175	14.9%	349	1.11 (0.60, 2.06)	0.743
BMI 30.0–40.0 kg/m^2	12/92	13.0%	18/93	19.4%	179	0.45 (0.21, 0.97)	0.040
**Educational level [c]**							
General secondary school	19/96	19.8%	26/116	22.4%	205	0.70 (0.46, 1.08)	0.110
Intermediate secondary school	52/342	15.2%	65/320	20.3%	645	0.67 (0.54, 0.83)	<0.001
High school	32/346	9.2%	18/335	5.4%	661	1.39 (0.87, 2.21)	0.172
**Parity**							
Primiparous	67/497	13.5%	53/418	12.7%	890	0.90 (0.78, 1.03)	0.136
Multiparous	36/288	12.5%	56/354	15.8%	622	0.70 (0.45, 1.08)	0.106

Abbreviations: BMI: body mass index; T1pp: assessment 6-8 weeks postpartum; T2pp: assessment one year postpartum; OR: odds ratio. [a] The total of participant numbers varies due to the applied covariates. [b] Binary logistic regression models fit using generalised estimating equations adjusted for pre-pregnancy BMI category, age, parity, baseline assessment and time interval between questionnaire completion date and birth date of the child. [c] General secondary school: general school which is completed through year 9; intermediate secondary school: vocational secondary school which is completed through year 10; high school: academic high school which is completed through year 12 or 13.
